# Supplementary figures and images for: Optimization of irrigation scheduling for maize in arid regions Northwest China based on water stress diagnosis in models
Source: PLoS One. 2026 Apr 17;21(4):e0344848. doi: 10.1371/journal.pone.0344848 (PMC13089687; doi:10.1371/journal.pone.0344848)

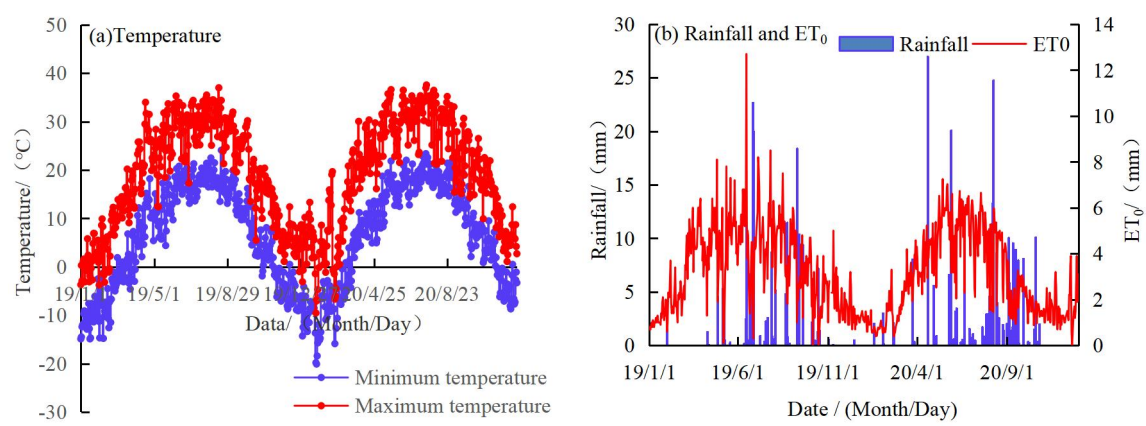

Figure 1 The meteorological parameters of the experimental area

Supplement: S1 Fig — (PDF) [file pone.0344848.s001.pdf]

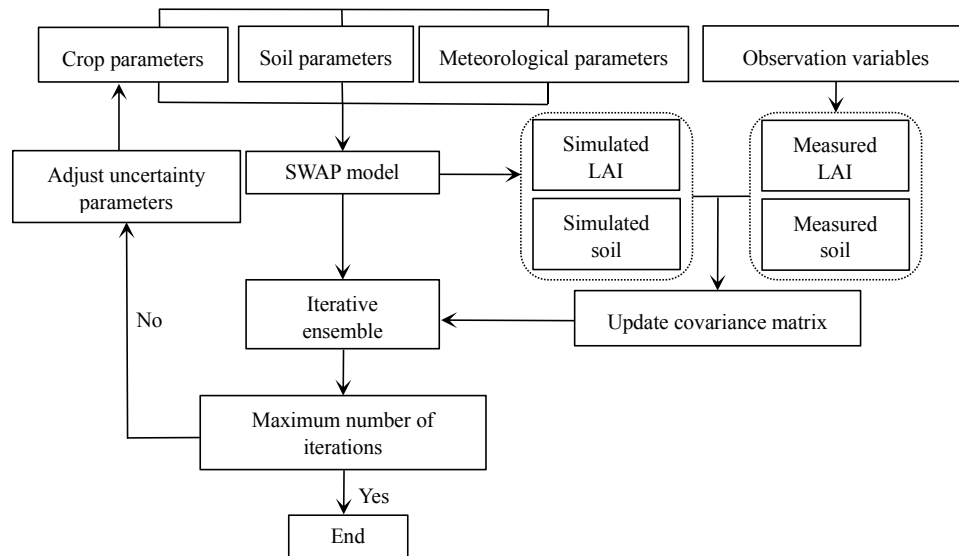

Figure 2 Flow chart of SWAP-IES assimilation system

Supplement: S2 Fig — (PDF) [file pone.0344848.s002.pdf]

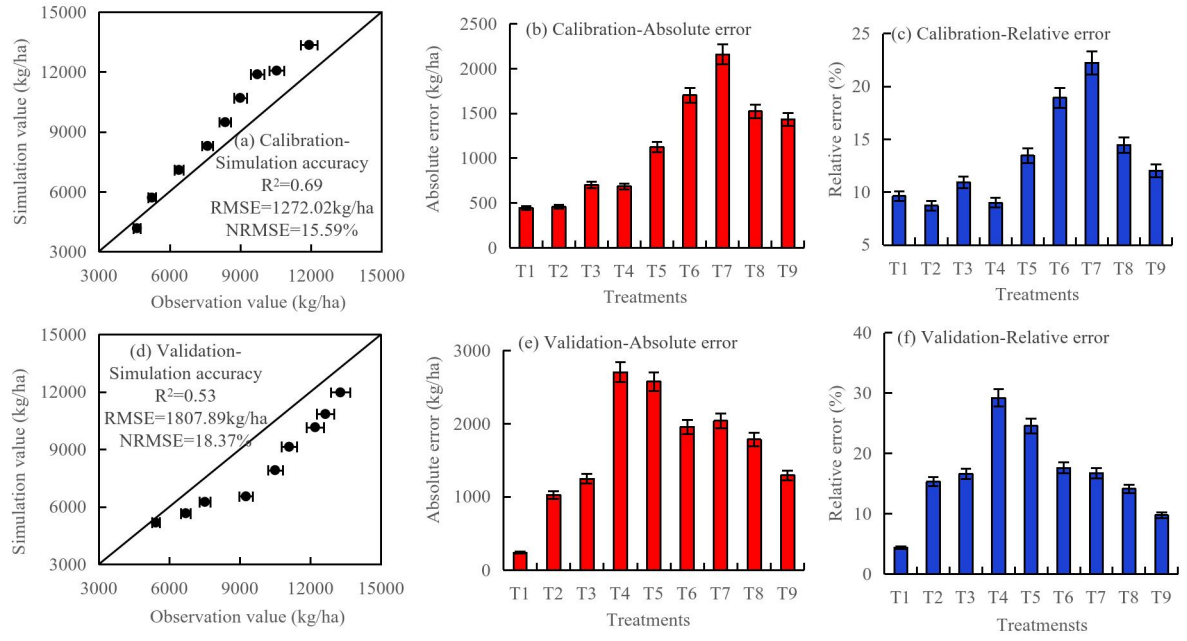

Figure 5 The simulation accuracy of maize yield during calibration and validation

Supplement: S5 Fig — (PDF) [file pone.0344848.s005.pdf]
