# Supplementary material for: Optimization of irrigation scheduling for maize in arid regions Northwest China based on water stress diagnosis in models
Source: PLoS One. 2026 Apr 17;21(4):e0344848. doi: 10.1371/journal.pone.0344848 (PMC13089687; doi:10.1371/journal.pone.0344848)
Supplement: S3 Fig — (PDF) [file pone.0344848.s003.pdf]

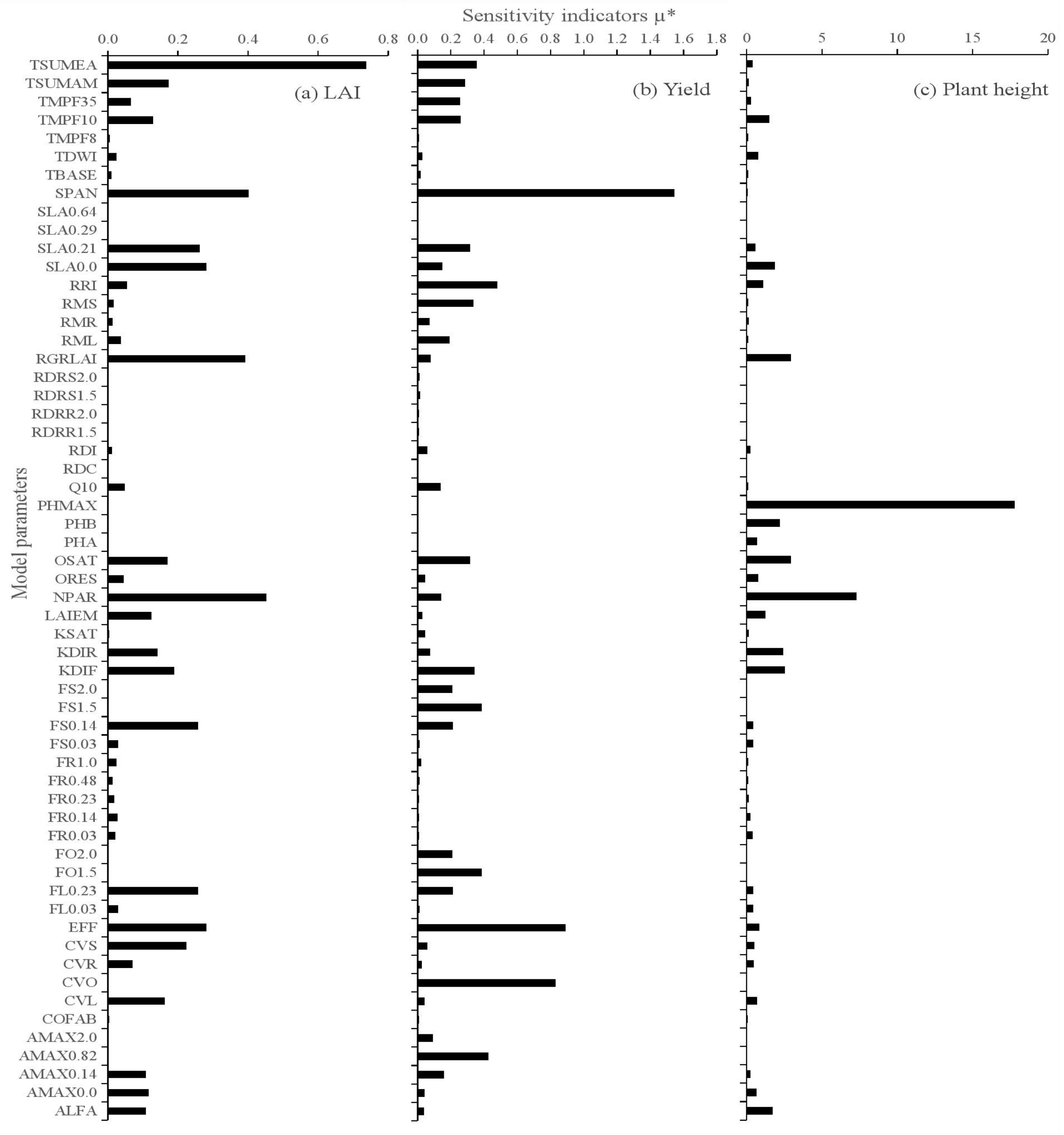

Figure 3 The sensitivity of maize LAI, plant height, and yield to the main parameters of the SWAP model
