# Supplementary material for: Optimization of irrigation scheduling for maize in arid regions Northwest China based on water stress diagnosis in models
Source: PLoS One. 2026 Apr 17;21(4):e0344848. doi: 10.1371/journal.pone.0344848 (PMC13089687; doi:10.1371/journal.pone.0344848)
Supplement: S4 Fig — (PDF) [file pone.0344848.s004.pdf]

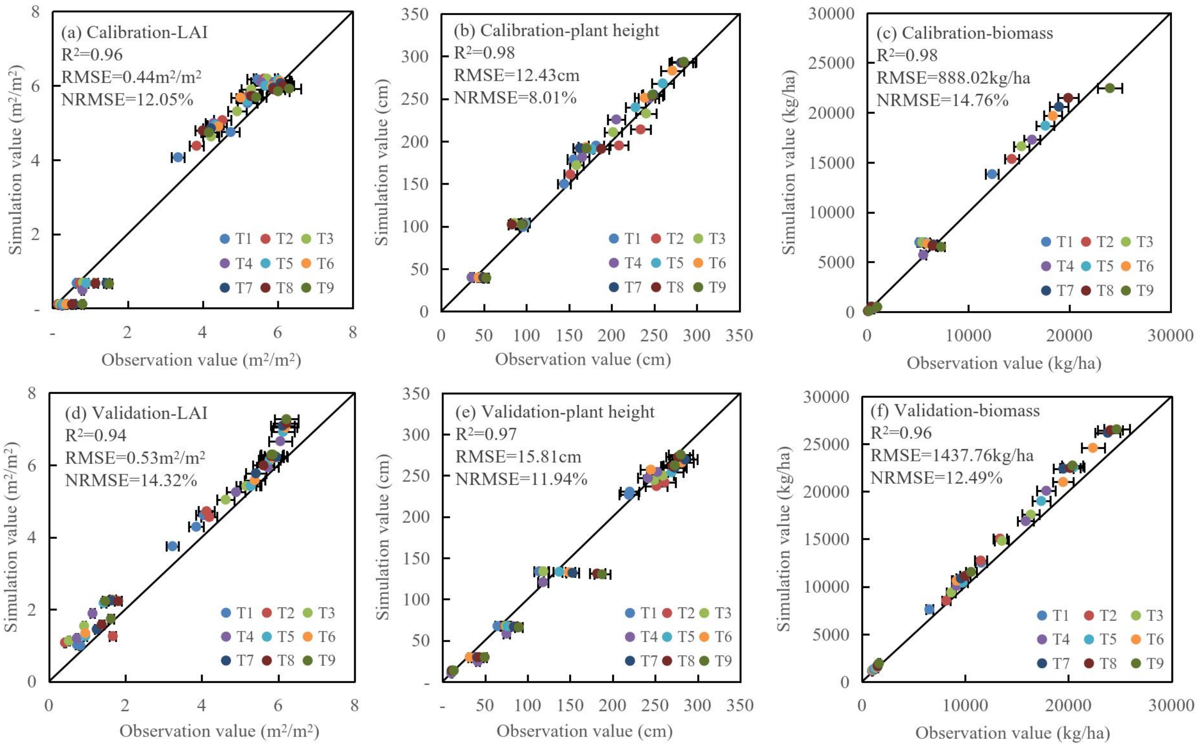

Figure 4 The simulation accuracy of maize LAI, plant height, and biomass during calibration and validation
