# Supplementary material for: Optimization of irrigation scheduling for maize in arid regions Northwest China based on water stress diagnosis in models
Source: PLoS One. 2026 Apr 17;21(4):e0344848. doi: 10.1371/journal.pone.0344848 (PMC13089687; doi:10.1371/journal.pone.0344848)
Supplement: S6 Fig — (PDF) [file pone.0344848.s006.pdf]

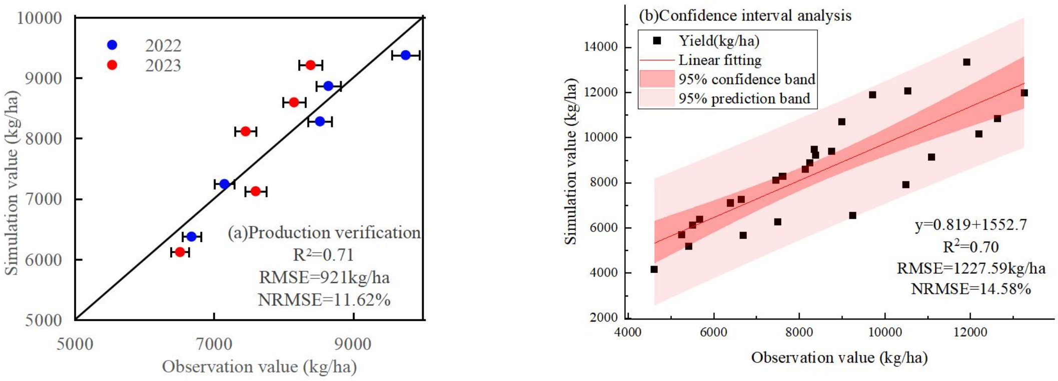

Figure 6 Simulation and confidence interval analysis of SWAP model on maize experimental yield
