# Supplementary material for: Optimization of irrigation scheduling for maize in arid regions Northwest China based on water stress diagnosis in models
Source: PLoS One. 2026 Apr 17;21(4):e0344848. doi: 10.1371/journal.pone.0344848 (PMC13089687; doi:10.1371/journal.pone.0344848)
Supplement: S8 Fig — (PDF) [file pone.0344848.s008.pdf]

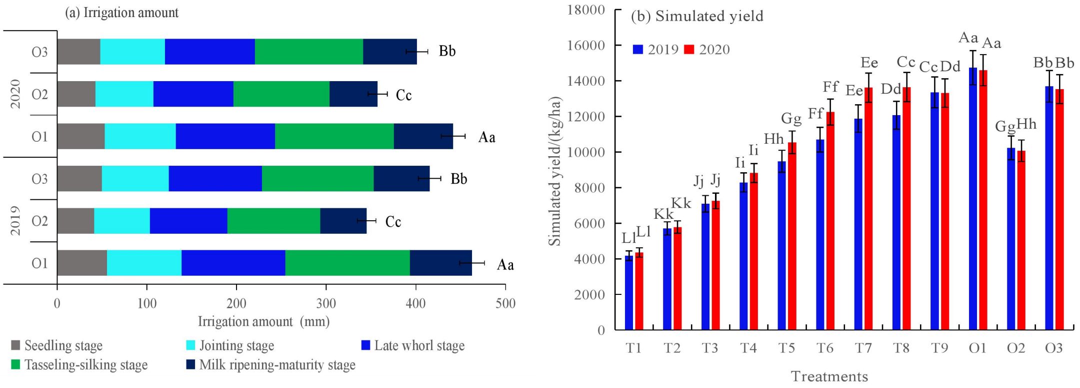

Figure 8 Optimized irrigation amount for each growth stage of maize and simulated yield, the analysis of variance in Figure 8a-b shows the results by year, upper case letters indicated 1% significance level, lower case letters indicated 5% significance level
