# Supplementary material for: Optimization of irrigation scheduling for maize in arid regions Northwest China based on water stress diagnosis in models
Source: PLoS One. 2026 Apr 17;21(4):e0344848. doi: 10.1371/journal.pone.0344848 (PMC13089687; doi:10.1371/journal.pone.0344848)
Supplement: S9 Fig — Upper case letters indicated 1% significance level, lower case letters indicated 5% significance level, and the blue fonts T1 - T9 and O1 - O3 below the data point were the number of processing or simulation scenarios. (PDF) [file pone.0344848.s009.pdf]

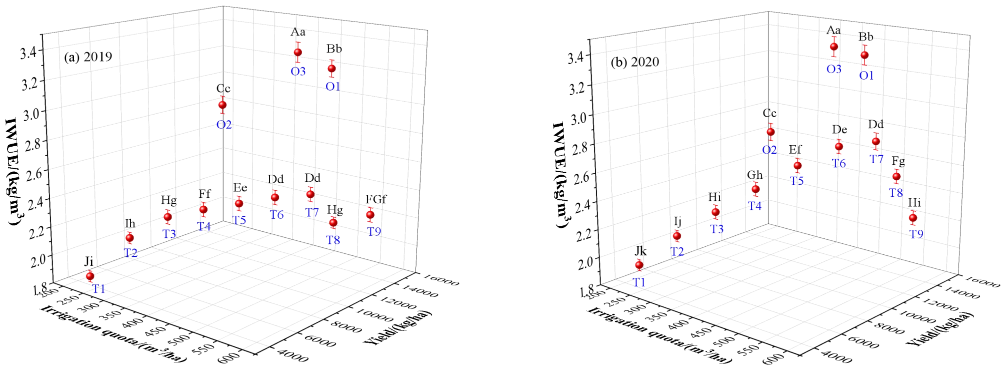

Figure 9 The irrigation water utilization efficiency (IWUE) of the optimized irrigation scheduling and the original irrigation scheduling. Upper case letters indicated 1% significance level, lower case letters indicated 5% significance level, and the blue fonts T1 - T9 and O1 - O3 below the data point were the number of processing or simulation scenarios
