# Supplementary material for: Optimization of irrigation scheduling for maize in arid regions Northwest China based on water stress diagnosis in models
Source: PLoS One. 2026 Apr 17;21(4):e0344848. doi: 10.1371/journal.pone.0344848 (PMC13089687; doi:10.1371/journal.pone.0344848)
Supplement: S1 Table — (PDF) [file pone.0344848.s010.pdf]

Table 1 Irrigation amount for each treatment at different growth stages of maize in Yongning (m^3/ha)

Treatments	Pre-sowing irrigation	Six leaf	Jointing		Late whorl	Tasseling	Silk emergence	Milk ripening	Total
T1	225	225	225	225	225	225	225	225	1800
T2	270	270	270	270	270	270	270	270	2160
T3	315	315	315	315	315	315	315	315	2520
T4	360	360	360	360	360	360	360	360	2880
T5	405	405	405	405	405	405	405	405	3240
T6	450	450	450	450	450	450	450	450	3600
T7	495	495	495	495	495	495	495	495	3960
T8	540	540	540	540	540	540	540	540	4320
T9	585	585	585	585	585	585	585	585	4680
