# Supplementary material for: Optimization of irrigation scheduling for maize in arid regions Northwest China based on water stress diagnosis in models
Source: PLoS One. 2026 Apr 17;21(4):e0344848. doi: 10.1371/journal.pone.0344848 (PMC13089687; doi:10.1371/journal.pone.0344848)
Supplement: S2 Table — (PDF) [file pone.0344848.s011.pdf]

Table 2 The range of Ws values for maize at different growth stages during the optimization process of irrigation scheduling

Reproductive stage		Seedling	Jointing	Late whorl	Tasseling-silk emergence	Milk ripening-maturity
The range of values for Ws	O1	0.95	0.95	0.95	0.95	0.95
	O2	0.90	0.90	0.90	0.90	0.90
	O3	0.90	0.95	0.95	0.95	0.95
