# Supplementary material for: Optimization of irrigation scheduling for maize in arid regions Northwest China based on water stress diagnosis in models
Source: PLoS One. 2026 Apr 17;21(4):e0344848. doi: 10.1371/journal.pone.0344848 (PMC13089687; doi:10.1371/journal.pone.0344848)
Supplement: S3 Table — (PDF) [file pone.0344848.s012.pdf]

Table 3 The simulation accuracy of maize LAI, plant height, and biomass for each treatments during calibration and validation

Years	Treat-ments	LAI			Biomass			Plant height		
		R^2	RMSE (m^2/m^2)	NRMSE (%)	R^2	RMSE (kg/ha)	NRMSE (%)	R^2	RMSE (cm)	NRMSE (%)
2019	T1	0.95	0.45	13.64	0.97	852.95	26.93	0.98	11.24	10.04
	T2	0.95	0.45	13.32	0.98	791.89	22.06	0.98	13	10.28
	T3	0.97	0.40	11.3	0.98	652.79	17.46	0.99	10.37	8.19
	T4	0.96	0.45	12.19	0.99	636.65	17.64	0.98	10.12	8.22
	T5	0.97	0.35	9.51	0.99	662.96	16.31	0.99	9.72	7.21
	T6	0.96	0.41	10.67	0.99	965.14	17.68	0.98	12.3	9.22
	T7	0.95	0.47	12.08	0.99	815.25	19.74	0.98	14.52	10.3
	T8	0.95	0.45	11.82	0.99	812.27	18.19	0.94	23.64	16.26
	T9	0.93	0.54	13.41	0.98	1031.19	24.75	0.93	27.01	18.15
2020	T1	0.93	0.42	14.45	0.94	1016.49	12.74	0.95	12.48	9.41
	T2	0.93	0.47	13.93	0.93	1066.01	12.21	0.96	12.13	8.51
	T3	0.95	0.47	12.12	0.98	962.23	8.95	0.97	11.7	7.71
	T4	0.95	0.52	12.33	0.97	1315.94	10.88	0.97	12.41	7.74
	T5	0.94	0.54	12.31	0.97	1358.13	10.18	0.99	9.63	5.72
	T6	0.94	0.53	11.91	0.97	1535.69	10.64	0.98	13.53	7.78
	T7	0.94	0.56	12.34	0.96	1986.33	13.01	0.97	15.22	8.63
	T8	0.94	0.54	11.63	0.97	1763.82	11.42	0.99	11.28	6.42
	T9	0.92	0.65	13.70	0.97	1586.74	10.11	0.98	12.77	7.25
