# Supplementary material for: Optimization of irrigation scheduling for maize in arid regions Northwest China based on water stress diagnosis in models
Source: PLoS One. 2026 Apr 17;21(4):e0344848. doi: 10.1371/journal.pone.0344848 (PMC13089687; doi:10.1371/journal.pone.0344848)
Supplement: S4 Table — (PDF) [file pone.0344848.s013.pdf]

Table 4 Irrigation amount for each treatment at different growth stages of maize in Pingluo (m^3/ha)

Treat-ments	Pre-sowing irrigation	Six leaf	Jointing		Late whorl	Tasseling	Silk emergence	Milk ripening	Total
S1	300	300	300	300	300	300	300	300	2400
S2	300	300	300	375	375	375	375	300	2700
S3	300	375	375	375	375	450	375	375	3000
S4	300	375	450	450	450	450	450	375	3300
S5	300	450	450	450	525	525	450	450	3600
