# Supplementary material for: Optimization of irrigation scheduling for maize in arid regions Northwest China based on water stress diagnosis in models
Source: PLoS One. 2026 Apr 17;21(4):e0344848. doi: 10.1371/journal.pone.0344848 (PMC13089687; doi:10.1371/journal.pone.0344848)
Supplement: S1 Appendix — (PDF) [file pone.0344848.s014.pdf]

Appendix 1 Main basic crop parameter values and sources of maize in SWAP model

Number	Parameter	definition	Value	Source
1	TSUMEA	Accumulated temperature from emergence to flowering/℃	822.0	Observation
2	TSUMAM	Accumulated temperature from flowering to maturity/℃	1252.75	Observation
3	TDWI	Biomass at emergence/（$kg \cdot ha^{-1}$）	40.0	Observation
4	LAIEM	Leaf area index at emergence/（$m^2 \cdot m^{-2}$）	0.026	Observation
5	SLA(0.0-0.3-2.0)	Specific Leaf Area/（$ha \cdot kg^{-1}$）	0.0016-0.002-0.002	Observation
6	SPAN	Leaf age at 35 ℃/（d）	38.0	Calibration
7	EFF	Light energy utilization efficiency of leaves/（$kgCO_2 \cdot J^{-1}$）	0.63	Calibration
8	K_{dif}	Scattering light extinction coefficient	0.60	Calibration
9	K_{dir}	Extinction coefficient of direct light	0.75	Calibration
10	AMAX0	Maximum CO_2 assimilation rate during seedling emergence/($kg \cdot ha^{-1} \cdot h^{-1}$)	70.0	Observation
11	AMAX1.5	Maximum CO_2 assimilation rate during flowering/($kg \cdot ha^{-1} \cdot h^{-1}$)	63.0	Observation
12	AMAX2	Maximum CO_2 assimilation rate during milk ripening/($kg \cdot ha^{-1} \cdot h^{-1}$)	40.0	Observation
13	RGRLAI	Daily maximum relative growth rate of leaf area index/（$ha \cdot ha^{-1}$）	0.051	Observation
14	CVL	Efficiency of assimilates transformation in leaf growth/($kg \cdot kg^{-1}$)	0.65	Calibration
15	CVO	Efficiency of assimilates transformation in ear growth/($kg \cdot kg^{-1}$)	0.71	Calibration
16	CVR	Efficiency of root growth assimilation conversion/($kg \cdot kg^{-1}$)	0.72	Default
17	CVS	Conversion efficiency of stem growth assimilates/($kg \cdot kg^{-1}$)	0.69	Default
18	TBASE	Lower limit of minimum temperature for seedling emergence/℃	8.0	Default
19	FR(0.0-1.1-2.0)	Distribution coefficient of dry matter to roots/($kg \cdot kg^{-1}$)	0.4-0.0-0.0	Default
20	FL(0.0-0.48-0.9-2.0)	Distribution coefficient of dry matter to leaves/($kg \cdot kg^{-1}$)	0.62-0.40-0.28-0.0	Default
21	FS(0.0-0.48-0.9-1.25-2.0)	Distribution coefficient of dry matter to stem/($kg \cdot kg^{-1}$)	0.38-0.6-0.72-0.0	Default
22	FO(0.0-1.25-2.0)	Distribution coefficient of dry matter to ear/($kg \cdot kg^{-1}$)	0.0-1.0-1.0	Default
23	Q10	Relative increase rate of respiratory rate for every 10 ° C increase in temperature	2.0	Default
24	RML	Leaves maintain respiratory rate/($kg \cdot kg^{-1} \cdot d^{-1}$)	0.03	Default
25	RMO	Store organs to maintain relative respiratory rate/($kg \cdot kg^{-1} \cdot d^{-1}$)	0.01	Default
26	RMR	Root relative maintenance of respiratory rate/($kg \cdot kg^{-1} \cdot d^{-1}$)	0.01	Default
27	RMS	Stem maintains relative respiratory rate/($kg \cdot kg^{-1} \cdot d^{-1}$)	0.015	Default
28	HLIM1	The upper limit of soil water potential when the root system can absorb water from the soil/cm	-10.0	Reference [1]
29	HLIM2U	The upper limit of soil water potential that is not affected by water stress for the water absorption term of the upper layer of soil roots/cm	-20.0	
30	HLIM2L	The upper limit of soil water potential that is not affected by water stress for all soil layer root water absorption items/cm	-20.0	
31	HLIM3H	The lower limit of soil water potential that is not affected by water stress on root water absorption under high air pressure/cm	-450.0	
32	HLIM3L	Lower limit of soil water potential under low-pressure conditions where root water uptake is not affected by water stress/cm	-550.0	
33	HLIM4	Soil water potential when root system stops absorbing water/cm	-10000.0	

[1] Yuan C. Simulation of water-salt transport and balance in cultivated-wasteland system based on SWAP model in Hetao Irrigation District of China. Agricultural Water Management. 2024; 305: 109132. https://doi.org/10.1016/j.agwat.2024.109132
