# Supplementary material for: Optimization of irrigation scheduling for maize in arid regions Northwest China based on water stress diagnosis in models
Source: PLoS One. 2026 Apr 17;21(4):e0344848. doi: 10.1371/journal.pone.0344848 (PMC13089687; doi:10.1371/journal.pone.0344848)
Supplement: Attachment — Submitted filename: Response to Reviewers.doc [file pone.0344848.s016.doc]

Dear editor:

Thank you and the two anonymous reviewers greatly for the insightful comments and suggestions on the early version of the manuscript. We have taken all of them seriously and had major revisions accordingly. The major modification is marked in blue. Your reconsiderations are highly appreciated!

Sincerely yours

Best Regards

Jin Jianxin

**Reviewer # 1 Questions and our responses**

**Question1:** The study applies the SWAP-IES system for irrigation optimization, but it is not entirely clear what is novel beyond integrating previously established methods. Similar work exists on combining crop models with data assimilation to optimize irrigation (eg, APSIM, DSSAT with Ensemble Kalman Filter/IES approaches). The authors should explicitly clarify:

- How their approach is innovative compared to previous SWAP-based irrigation optimization studies. Please cite and use the following papers (https://doi.org/10.1016/j.agwat.2025. 109668;<https://doi.org/>10.3390/w14223647; https://doi.org/10.1016/j.agwat.2021.107122)

**Response:** We appreciate the meticulous review by the expert reviewers. At present, there are several research reports on optimizing irrigation scheduling via crop growth assimilation simulation systems, and excellent results have been attained in various research areas. The main innovation of the method proposed in this manuscript, compared with other methods, lies in：（1）The Iterative Ensemble Smoother (IES) algorithm employed in this study is relatively advanced. It can achieve global exploration by leveraging iteration and ensemble diversity while needing a limited computational cost. Additionally, it exhibits robustness against strong nonlinearity.（2）By employing the SWAP-IES system to simulate the water stress status of maize, dynamically adjusting the irrigation scheduling, and verifying the results with data from simulated years, researchers not only improve the simulation accuracy of maize growth but also significantly enhance the accuracy of irrigation scheduling. The use of water stress coefficient (actual transpiration/potential transpiration of maize) as an indicator for determining irrigation greatly improves water use efficiency compared to traditional irrigation based on soil moisture. The above innovative points (2) and (3) have been supplemented in the introduction section, and (1) has been supplemented in the 1.2 SWAP-IES system construction section, with the modified parts highlighted in blue font. The main references are as follows:

[1]Attia A, Govind A, Qureshi AS, Feike T, Rizk MS, Shabana MMA, Kheir AMS. Coupling process-based models and machine learning algorithms for predicting yield and evapotranspiration of maize in arid environments. Water. 2022; 14(22): 3647. https://doi.org/ 10.3390/w14223647

[2]Kheir AMS, Alrajhi AA, Ghoneim AM, Ali EF, Magrashi A, Zoghdan MG, Abdelkhalik SAM, Fahmy AE, Elnashar A. Modeling deficit irrigation-based evapotranspiration optimizes wheat yield and water productivity in arid regions. Agricultural Water Management, 2021, 256: 107122. https://doi.org/10.1016/j.agwat. 2021.107122

[3]Elsayed ML, Elkot AF, Noreldin T, Richard B, Qi A, Shabana YM, Saleh SM, Fitt BDL, Kheir AMS, Kheir AM. Optimizing wheat yield and water productivity under water scarcity: A modeling approach for irrigation and cultivar selection across different agro-climatic zones of Egypt. Agricultural Water Management. 2025; 317: 109668. https://doi.org/10.1016/j.agwat. 2025.109668

[4]Li X, Zhang J, Cai X, Huo ZL, Zhang CL. Simulation-optimization based real-time irrigation scheduling: A human-machine interactive method enhanced by data assimilation. Agricultural Water Management. 2023; 276: 108059.https://doi.org/10.1016/j.agwat.2022. 108059

[5]Wang Y, Huang D, Zhao L, Shen HZ, Xing XG, Ma XY. The distributed CERES-Maize model with crop parameters determined through data assimilation assists in regional irrigation schedule optimization. Computers and Electronics in Agriculture. 2022; 202: 107425. https://doi.org/10.1016/j.compag.2022.107425

**Question2:** Whether the Ws-based irrigation thresholds (O1-O3) are new or adapted from literature, and if adapted, how they are improved here.

**Response:** We appreciate the meticulous review by the expert reviewers. The three irrigation thresholds set in the manuscript were mainly based on the water demand pattern during the growth period of maize, with the principle of no water stress or mild water stress. Irrigation was carried out when the transpiration rate decreased to 0.9 or 0.95 of the potential transpiration rate, in order to maximize maize yield as much as possible. Relevant explanations had been added to the section on irrigation system optimization strategies in 1.3. The added content was as follows：

“In order to achieve precise irrigation, the water stress coefficient Ws was used to control the amount and time of irrigation. The irrigation principle was that no water stress or only mild water stress occurred in each growth stage of maize. Ws was set at two lower limits of 0.9 and 0.95, and the irrigation trigger threshold for each growth stage was set according to the water demand pattern of maize. The irrigation amount was determined by irrigating to a non-stress state (i.e., Ws = 1). In order to facilitate field management and avoid frequent irrigation operations, three irrigation decision gradients were set up for simulation calculation, as shown in Table 2.”

**Question3:** Calibration/validation results for LAI, biomass, and plant height are strong, but yield simulation accuracy is only moderate (R²=0.53–0.69, with large RMSE). This weak yield performance should be addressed. Discuss potential causes (parameterization, model structural limits, lack of physiological constraints, or field variability). Consider using multi-year or cross-site calibration to improve robustness. Quantify uncertainty more rigorously (eg, confidence intervals, bootstrapping).

**Response:** We appreciate the meticulous review by the expert reviewers. Due to the fact that LAI was mainly used as the standard for global optimization during the model parameter calibration process, some treatments may have led to overfitting of LAI. At the same time, the SWAP model lacked consideration of stress factors such as soil salinization, spatial variability of soil properties, pests and diseases, weeds, etc. in the simulation process. In addition, the model itself assumed parameterization or linearization when describing the growth mechanism of maize. These factors combined resulted in low simulation accuracy of the model for some treatments of mazie yield (such as T4 - T7 in the model calibration process and T4 - T6 in the validation process). Therefore, based on the review comments, the SWAP model was revalidated using maize irrigation experiments conducted in Pingluo County, Ningxia, China, from 2022 to 2023 (only for yield validation). At the same time, confidence interval analysis was conducted on the measured and simulated yields of the experiments in Yongning County and Pingluo County. It was found that all simulated values were within the 95% prediction band, and most simulated values were within the 95% confidence band. The calibrated SWAP model parameters have a certain degree of scientific validity and reliability. In Section 2.2.2 of the main text, the simulation of yield indicators has been supplemented with an analysis of the low consistency of yield simulation. The specific supplementary content is as follows:

“Due to the model's failure to account for the impacts of various stress factors, such as soil salinity, spatial variability of soil properties, weeds, pests, and diseases, on yield, as well as issues related to parameter calibration, including heteroscedasticity and overfitting, along with the model's assumptions regarding the parameterization of maize growth processes, the model could not achieve a high degree of simulation accuracy for the maize yields of all treatments. Consequently, this led to significant errors in some simulated yield values and a reduction in the overall simulation consistency for the yields of all treatments. To verify the model's reliability in yield simulation, it was necessary to conduct multi-point and multi-year field experiments to further assess the model's stability.”

Section 2.2.3 has been added to the manuscript, which mainly aims to introduce the reliability of the SWAP model in simulating maize yield across multiple sites and over multiple time periods. The main supplementary content is as follows:

2.2.3 Independent validation and confidence interval analysis

(1) Overview of the experimental area and experimental design

The simulation accuracy of the SWAP model for maize yield was further validated using the irrigation experiment conducted in Pingluo County, Ningxia from 2022 to 2023. The experimental area was located in Baofeng Town (E106 ° 43 ′, N39 ° 02 ′), with a temperate continental climate. The average annual rainfall was 177 mm, the average annual evaporation was 1755 mm, the average annual sunshine hours were 3008 h, and the frost-free period was 171 d. The soil in the experimental area was sandy loam, having an average field water holding capacity of 27 % and an average bulk density of 1.48 g/cm^3^. The available nitrogen, phosphorus, and potassium contents, as well as the organic matter content in the cultivated layer of the experimental site, are 74.36 mg/kg, 13.21 mg/kg, 114.28 mg/kg, and 1.9%, respectively. The experiment was a single-factor experiment on irrigation amount, using drip irrigation as the irrigation method. Five gradient irrigation amounts were set up with the conventional irrigation amount of local farmers as the control, and the irrigation quotas were 2400 m^3^/ha (S1), 2700 m^3^/ha (S2), 3000 m^3^/ha (S3), 3300 m^3^/ha (S4), 3600 m^3^/ha (S5). The division of experimental plots, fertilization, indicator determination methods, and other field management measures were the same as before. The irrigation amount for each growth period is shown in Table 4.

Table 4 Irrigation amount for each treatment at different growth stages of maize in Pingluo (m^3^/ha)

| Treatments | Pre-sowing irrigation | Six leaf | Jointing | | Late whorl | Tasseling | Silk emergence | Milk ripening | Total |
| --- | --- | --- | --- | --- | --- | --- | --- | --- | --- |
| S1 | 300 | 300 | 300 | 300 | 300 | 300 | 300 | 300 | 2400 |
| S2 | 300 | 300 | 300 | 375 | 375 | 375 | 375 | 300 | 2700 |
| S3 | 300 | 375 | 375 | 375 | 375 | 450 | 375 | 375 | 3000 |
| S4 | 300 | 375 | 450 | 450 | 450 | 450 | 450 | 375 | 3300 |
| S5 | 300 | 450 | 450 | 450 | 525 | 525 | 450 | 450 | 3600 |

(2) Validation results and confidence interval analysis

The simulation and confidence-interval analysis results of the SWAP model for maize experimental yield were presented in Figure 6. The analysis revealed that the SWAP model had a simulated R^2^ of 0.71, an RMSE of 921 kg/ha, and an NRMSE of 11.62% for maize yield in Pingluo County (Figure 6a), indicating good simulation accuracy. Through the confidence-interval analysis of the measured and simulated values of all yield in Yongning County and Pingluo County (Figure 6b), it was observed that many yield values fell within the 95% confidence band, and all yield values were included in the 95% prediction band, with an R^2^ of 0.70, an RMSE of 1227.59 kg/ha, and an NRMSE of 14.58%. Overall, the simulation accuracy of the SWAP model for maize yield could meet general needs and had certain applicability. Therefore, the established maize SWAP model parameter set had a certain degree of scientificity and reliability.

Figure 6 Simulation and confidence interval analysis of SWAP model on maize experimental yield

**Question4:** The choice of Ws thresholds (0.90-0.95) seems arbitrary. There is no clear agronomic justification or sensitivity analysis showing why these values were selected above other possible thresholds. Suggest performing a sensitivity analysis to demonstrate the effect of varying Ws thresholds on yield, IWUE, and total irrigation.

**Response:** We appreciate the meticulous review by the expert reviewers. The basic principle for optimizing the irrigation strategy in this study was as follows: to keep maize free from water stress or only under mild water stress. Therefore, the lower irrigation limit, Ws, was set to 0.9 or 0.95, and different values were assigned to it throughout the entire growth period based on the water requirement pattern of maize. After reviewing relevant literature and analyzing model parameters, it was found that the soil moisture content at which root water uptake is affected by water stress is approximately equal to the field capacity. The corresponding parameter HLIM3L, which represents a relevant parameter, was -450 cm. Thus, when Ws was set to 0.9 and 0.95, the corresponding soil moisture content ranged from 80% to 100% of the field capacity. Additionally, the authors had previously measured the soil water characteristic curves at different depths in the experimental area (see the figure below). The results showed a good correlation between the soil negative pressure value and the water stress coefficient Ws. The soil negative pressure value is the critical value for root water uptake recommended in the literature (cited in this manuscript). Consequently, Ws was set to 0.95 or 0.90 to ensure no water stress on maize, with the primary objective of proposing an irrigation regime characterized by high yield and high irrigation water use efficiency.

Fig.1 Soil pressure film instrument Fig.2 Soil moisture characteristic curves of each soil layer

**Question5:** The authors acknowledge that the optimized schedules have not been field-tested. This is a significant limitation.

**Response:** We appreciate the meticulous review by the expert reviewers. The manuscript simulated the water-stress conditions that maize experiences during field experiments, adjusted the irrigation time and amount planned for the experiment, and proposed an optimized irrigation scheduling, which significantly improved maize yield and water-use efficiency. **The focus of the manuscript is to propose a method for irrigation decision-making based on the water-stress coefficient Ws through simulation analysis**. Although it has not been tested in field experiments, theoretically, maize transpiration is an important indicator reflecting water stress. Models such as SWAP use this indicator to characterize the impact of water on maize growth. Irrigation based on the actual transpiration of maize can minimize the impact of water stress and improve water-use efficiency. The proposed irrigation decision-making method has a certain degree of scientificity and reliability, and it will be validated and improved in subsequent research to further optimize the irrigation decision-making method and achieve precise and efficient irrigation.

**Question6:** The discussion includes some literature comparison, but the differences in irrigation amounts between this study and others (eg, APSIM/DSSAT outputs) need deeper analysis. Are the differences due to model choice, parameterization, soil/meteorological conditions, or the inclusion of salinity leaching requirements in this region?

**Response:** We appreciate the meticulous review by the expert reviewers. Based on the review comments, we compared the maize irrigation amounts optimized by previous models and analyzed the internal reasons for the differences in irrigation amounts from the perspectives of optimization objectives, boundary conditions, parameter settings, and so on. The main supplementary content is as follows:

“These research results differed significantly from the irrigation amount proposed in this article, mainly because the optimization of maize irrigation scheduling in different regions was often constrained by local water resource conditions, field management practices, and climatic factors. Consequently, the simulation process differed in terms of constraint settings, cost functions, and optimization objectives. For instance, in water-scarce areas, irrigation quotas, water-use efficiency, or actual evapotranspiration (ET_a_) were usually treated as the primary constraints, even if this entailed a certain degree of yield loss. In areas with convenient field management, both the number of irrigation events and the irrigation amount were typically set as variables; suitable irrigation scheduling was identified through extensive simulations in a two-dimensional space. In regions with mechanized harvesting, parameters related to the maize growth period were adjusted to maximize rainfall utilization and reduce irrigation water demand, thereby developing water management strategies that balanced rainfall and artificial irrigation. Additionally, the maize irrigation scheduling proposed in these studies often included additional conditions such as adjustments to sowing dates and allowances for yield loss.”

**Question7:** The Yellow River Irrigation Area has salinity challenges, yet the model does not appear to incorporate salinity effects in simulating yield or water stress.
**Response:** We appreciate the meticulous review by the expert reviewers. As the reviewer pointed out, this study did not consider the impact of salt stress on maize growth during the simulation process because salt alkali was not the main stress factor in the experimental area. The proposed irrigation amount for maize in this study was relatively large, mainly because the simulation goal was to minimize water stress or only allow for mild water stress on maize. The relevant discussion had been modified in the discussion section, as follows:

“Because the irrigation amount proposed in study was based on the assumption that maize did not experience water stress or only experienced mild water stress, the aim was to minimize the loss of maize yield due to water stress. From this, it could be seen that the irrigation scheduling for maize had strong regional and purposive characteristics. Reasonable irrigation scheduling was developed based on regional characteristics and management requirements to achieve multiple goals of improving maize yield and water use efficiency.”

**Question8:** No statistical significance testing (eg, ANOVA) is provided for differences in yield or IWUE among treatments, which limits confidence in the claims of superiority for O1–O3

**Response:** We appreciate the meticulous review by the expert reviewers. According to the review comments, an inter-group analysis of variance was conducted on the simulation results of different treatments and O1-O3, and the main text was revised. The analysis found that the yield of each simulated scenario showed significant differences (P<0.01), but the irrigation water use efficiency presented a more complex situation. Among them, some T1-T9 treatments had insignificant differences in their simulation results, but the O1-O3 groups showed significant differences. This indicated that different irrigation lower limits at different growth stages of maize had a significant impact on irrigation water use efficiency. The modified parts of the main text were marked in blue font.

**Question9:** Figures 6 and 7 (Ws patterns) are overly complex, and the color scheme and legend are difficult to interpret.

**Response:** We appreciate the meticulous review by the expert reviewers. The SWAP-IES system proposed in this study achieved data assimilation calculation through iterative optimization and set prediction. Its core steps were as follows:

①Several sample sets of the model parameters to be calibrated were randomly generated, and the sample size was set according to computer hardware equipment, calculation consumption time, etc. ②Each set of model parameters to be calibrated was combined with other fixed parameters to generate a series of new model parameter sets. The SWAP model was run separately to generate a series of simulation processes for maize growth (including LAI, plant height, biomass, soil moisture, yield, etc.). ③The simulated growth indicators of maize were compared with the growth indicators measured in field experiments. The covariance between the two was calculated, and the Kalman gain was computed using this covariance. The Kalman gain was then used to adjust the model's parameters to be corrected until the preset convergence standard or maximum iteration number was reached. ④The average of all parameter set simulation results was calculated as the final simulation result. From this, it could be seen that a large number of simulation processes were generated during the simulation, with the same number as the sample size set in the first step. The gray areas in Figure 6 and Figure 7 represented the coverage range of all sample simulation processes. To clearly show the water stress situation of maize in the model calibration and validation process for each treatment, only the average value of the water stress coefficient Ws was retained in the figure, and the gray coverage area was removed. The modified figure is as follows:

Figure 7 The water stress coefficients (Ws ) of each treatment during the model calibration and verification, the values in the figure represent the mean values simulated for each ensemble

**Question10:** Figures 8 and 9 should include statistical error bars and significance groupings.

**Response:** We appreciate the meticulous review by the expert reviewers. According to the review comments, a variance analysis was conducted on the maize yield and irrigation water use efficiency under different treatment and simulation scenarios in Figures 8 and 9. Error lines and significant difference markers were drawn. Please refer to Question 8 for specific modifications.

**Reviewer # 2 Questions and our responses**

**Question1:** In the title, you need to clarify the geographical location of the study at least the country name.

**Response:** We appreciate the meticulous review by the expert reviewers. This study was conducted in the arid inland areas of northwest China, with the main objective of developing an irrigation strategy that could dynamically adjust according to the timely water stress status of maize. The main research content included: (1) using the Morris sensitivity analysis method to analyze the sensitivity of various parameters of the SWAP model to the output results, and proposing the sensitive parameters that needed to be checked in detail; (2) calibrating and validating the sensitive parameters of the SWAP model based on field irrigation experiment data from 2019 - 2020; (3) simulating and analyzing the water stress status of maize during the model calibration and validation process using the water stress coefficient Ws; (4) based on the simulation of water stress, adjusting the irrigation system and proposing irrigation strategies with high yield and irrigation water utilization efficiency. The manuscript title had been changed to“Optimization of Irrigation Scheduling for Maize in Arid Regions Northwest China Based on Water Stress Diagnosis in Models”.

**Question2:** (1) In line no. 9, you need to correct the word "was" by the word "is".

(2)In line no. 35, you need to substitute the word "effects" with the word "impacts" as it has more relation with climate conditions.

(3)In line no. 36, please remove the repeated "had" and complete the sentence by mentioning the good effect of irrigation on what?

**Response:** We appreciate the meticulous review by the expert reviewers. According to the review comments, the above wording errors were corrected. At the same time, in line 36, the manuscript mainly aimed to explain the use of irrigation in agricultural production to resist the adverse effects of drought climate on crop growth and ensure stable grain yield. The sentence was revised as follows:”For a long time, people had been able to resist the adverse impacts of climate conditions through irrigation, achieved high yields, and ensured stable food production”

**Question3:** (1)In line no. 116, please add the word "sowing" after "The" at the beginning of the line, then correct the phrase "maize sowing plants" to be "maize sown plants".

(2)In line no. 122, please put "in" instead of "with".

(3)In line no. 131, you need to correct the sentence to be "Additional testing has been done after rainfall, which is .....". Please mention the testing type or method.
**Response:** We appreciate the meticulous review by the expert reviewers. According to the review comments, the above wording errors were corrected. The 131st line described the use of the method mentioned previously to determine soil moisture content after rainfall. This sentence was revised to ”Additional testing has been done after rainfall, which is to determine the soil moisture content once according to the aforementioned method.”

**Question4:** (1) In line no. 132, please clarify at which age the plant height has been measured, and how frequent measurements were done.

(2)In line no. 138, how many measurements have been taken?

**Response:** We appreciate the meticulous review by the expert reviewers. The height and biomass of maize plants were measured at each growth stage, resulting in a total of 5 measurements throughout the entire growth period. The measurement period and the number of measurements throughout the entire growth period were added on line 132, and the specific modification is "Plant height and biomass were to be measured once at each growth stage of maize, and a total of 5 measurements were taken throughout the entire growth period."

**Question5:** (1)In line no. 139, please put after":" the beginning of the sentence which is "It was...".

(2)In line no. 142, put the "physiological maturity" instead of "maturity".

(3)In line no. 143, put "cm" after the number "10".
**Response:** We appreciate the meticulous review by the expert reviewers. According to the review comments, the above wording errors were corrected. In line 143, the number 10 indicates a spacing of 10 rows between each sampling point. This means that for the production measurement process, sample points are selected at intervals of 10 rows.

**Question6:** (1)In line no. 144, correct the sentence to be "and plants and ears were counted".

(2)In line no. 148, please correct the sentence to be "...and it was converted into the yield (kg) per hectare.

(3)In line no. 151, please remove "The" at the beginning and put "is" instead of "was".

(4)In line no. 162, make the "a" for "accumulated" in lower case.

(5)In line no. 163, after ":" put "They were"

(6)In line no. 427, after 20.22% write "in IWUE".

(7)In line no. 491, add the location at the end of the sentence.

**Response:** We appreciate the meticulous review by the expert reviewers. According to the review comments, the above wording errors were corrected. This experiment was conducted in the arid region of northwest China. The SWAP model was calibrated using water stress tests, and the SWAP - IES system was used to diagnose the water stress status of maize treated in these experiments. A method for dynamic optimization of maize irrigation strategy was proposed. The focus and main innovation of this manuscript were to propose a dynamic optimization method for the irrigation scheduling, providing reference and application for maize production in arid regions.
